# Tracing the possible evolutionary trends of *Morganella morganii*: insights from molecular epidemiology and phylogenetic analysis

**DOI:** 10.1128/msystems.00306-24

**Published:** 2024-06-17

**Authors:** Jiawei Chen, Yun Wu, Ge Zhang, Wei Kang, Tong Wang, Jin Li, Menglan Zhou, Li Zhang, Yong Liu, Xuesong Xu, Xinmiao Jia, Yingchun Xu, Yali Liu

**Affiliations:** 1 Department of Laboratory Medicine, State Key Laboratory of Complex Severe and Rare Diseases, Peking Union Medical College Hospital, Chinese Academy of Medical Sciences and Peking Union Medical College, Beijing, China; 2 Graduate School, Peking Union Medical College, Chinese Academy of Medical Sciences, Beijing, China; 3 Department of Clinical Laboratory, Shengjing Hospital of China Medical University, Shenyang, China; 4 Department of Clinical Laboratory, China-Japan Union Hospital, Jilin University, Changchun, China; 5 Center for Bioinformatics, National Infrastructures for Translational Medicine, Institute of Clinical Medicine & Peking Union Medical College Hospital, Chinese Academy of Medical Sciences and Peking Union Medical College, Beijing, China; Southern Medical University, Guangzhou, Guandong, China

**Keywords:** *Morganella morganii*, evolution, antimicrobial resistance genes, virulence-related genes, *bla*_KPC-2_-bearing plasmid

## Abstract

*Morganella morganii*, encompassing two subspecies, *subsp. morganii* and *subsp. sibonii*, is a common opportunistic pathogen, notable for intrinsic resistance to multiple antimicrobial agents. Despite its clinical significance, research into the potential evolutionary dynamics of *M. morganii* remains limited. This study involved the analysis of genome sequences from 431 *M*. *morganii* isolates, comprising 206 isolates that cause host infections, obtained from this study and 225 from the NCBI genome data sets. A diverse array of antimicrobial resistance genes (ARGs) was identified in *M. morganii* isolates, including *mcr-1*, *tet*(X4), *tmexCD-toprJ*, and various carbapenemase genes. In addition, a novel *bla*_KPC-2_-bearing plasmid with demonstrated conjugative capability was discovered in *M. morganii*. The majority of virulence-related genes (VRGs), except for the *hlyCABD* gene cluster, were found in almost all *M. morganii*. Three novel genospecies of *M. morganii* were identified, designated as *M. chanii*, *M. variant1*, and *M. variant2*. Compared to *M. sibonii*, *M. chanii* genospecies possessed a greater number of flagellar-related genes, typically located within mobile genetic elements (MGEs), suggesting potential for better environmental adaptability. Phylogenetic analysis further disclosed that *M. morganii* was divided into 12 sequence clusters (SCs). Particularly, SC9 harbored an elevated abundance of ARGs and VRGs, mainly toxin-related genes, and was associated with a higher presence of MGEs compared to non-SC9 strains. The collective findings suggest that *M. morganii* undergoes evolution driven by the influence of MGEs, thereby significantly enhancing its adaptability to selective pressures of environmental changes and clinical antimicrobial agents.

**IMPORTANCE:**

The growing clinical significance of *Morganella morganii* arises from its abundant virulence factors and antimicrobial resistance genes, resulting in elevated infection rates and increased clinical scrutiny. However, research on the molecular epidemiology and evolutionary trends of *M. morganii* has been scarce. Our study established a list of virulence-related genes (VRGs) for *M. morganii* and conducted a large-scale epidemiological investigation into these VRGs. Based on genomic classification, three novel genotypes of *M. morganii* were identified, representing evolutionary adaptations and responses to environmental challenges. Furthermore, we discovered the emergence of a sequence cluster enriched with antimicrobial resistance genes, VRGs, and mobile genetic elements, attributed to the selective pressure of antimicrobial agents. In addition, we identified a novel conjugative plasmid harboring the *bla*_KPC-2_ gene. These findings hold significance in monitoring and comprehending the epidemiology of *M. morganii*.

## INTRODUCTION

*Morganella morganii*, a facultative anaerobic Gram-negative bacterium, is ubiquitously found in environmental habitats and the gastrointestinal tracts of humans, mammals, and reptiles (1). *M. morganii* is categorized into two subspecies, *M. morganii subsp. sibonii* and *M. morganii subsp. morganii*, according to trehalose fermentation ability which is linked to the presence of the trehalose operon (*treR*, *treB*, and *treP*) (2). *M. morganii* is recognized as a significant opportunistic pathogen, frequently identified as a causative agent of nosocomial infections in adults, notably contributing to cases of urinary tract and wound infections during clinical observations (3). The mortality of *M. morganii* infections remained elevated in reported cases. A prior study showed a total of 150 out of 709 patients (21.2%) succumbed within 30 days following *M. morganii* bloodstream infections (BSIs) (4).

*M. morganii* possesses intrinsic resistance to a broad spectrum of antimicrobial agents including ampicillin, amoxicillin, and most of the first- and second-generation cephalosporins, attributed to chromosomally encoded *bla*_AmpC_ (5) as well as macrolides, lincosamides, glycopeptides, fosfomycin, fusidic acid, and colistin (6). Akin to other Enterobacterales, *M. morganii* also harbors a diverse array of acquired antimicrobial resistance genes (ARGs) such as *bla*_KPC-2_, *bla*_NDM_, *tet*(A), and *aadA1*, facilitated by the substantial presence of mobile genetic elements (MGEs) (7). Moreover, various virulence-related genes (VRGs) such as urease, iron acquisition systems, IgA protease, and hemolysins were identified in *M. morganii*, characterizing its ability to colonize diverse hosts. Notably, comparative genome analysis revealed that unique VRGs such as *eut* operon were carried by the *M. morganii* genome compared to other *Proteeae* members (8). Therefore, *M. morganii* has been labeled an emerging “superbug.” However, up to this point, there has been a lack of large-scale and long-term investigation into the carriage of VRGs in *M. morganii*.

Molecular epidemiology plays an integral role in understanding pathogenic bacteria, offering insights into their transmission routes, virulence, drug resistance, genetic variations, and evolutionary trends. Among various molecular techniques, multilocus sequence typing (MLST) stands out as a versatile and universally applicable sequence-based methodology, instrumental in elucidating clonal relationships among bacterial species (9). Regrettably, the MLST typing method has not yet been established for *M. morganii*. Currently, only a limited number of studies have conducted genomic epidemiology analysis on *M. morganii* using whole-genome data. Jing et al. developed a classification system based on genome sequencing, which categorized *M. morganii* into four distinct genospecies: *M. morganii*, *M. chanii*, *M. sibonii*, and *M. laugraudii* (7). However, the genomic characteristics of these individual genospecies were not delved into in detail. This study conducted the molecular epidemiological analysis of *M. morganii* including 206 isolates from this study and 225 isolates from the NCBI genome data sets and aimed to meticulously examine the characteristics of different sequence clusters, thereby elucidating the evolution of the *M. morganii* genome.

## RESULTS

### The sources and antimicrobial resistance of *Morganella morganii* collected in this study

**Fig 1:**
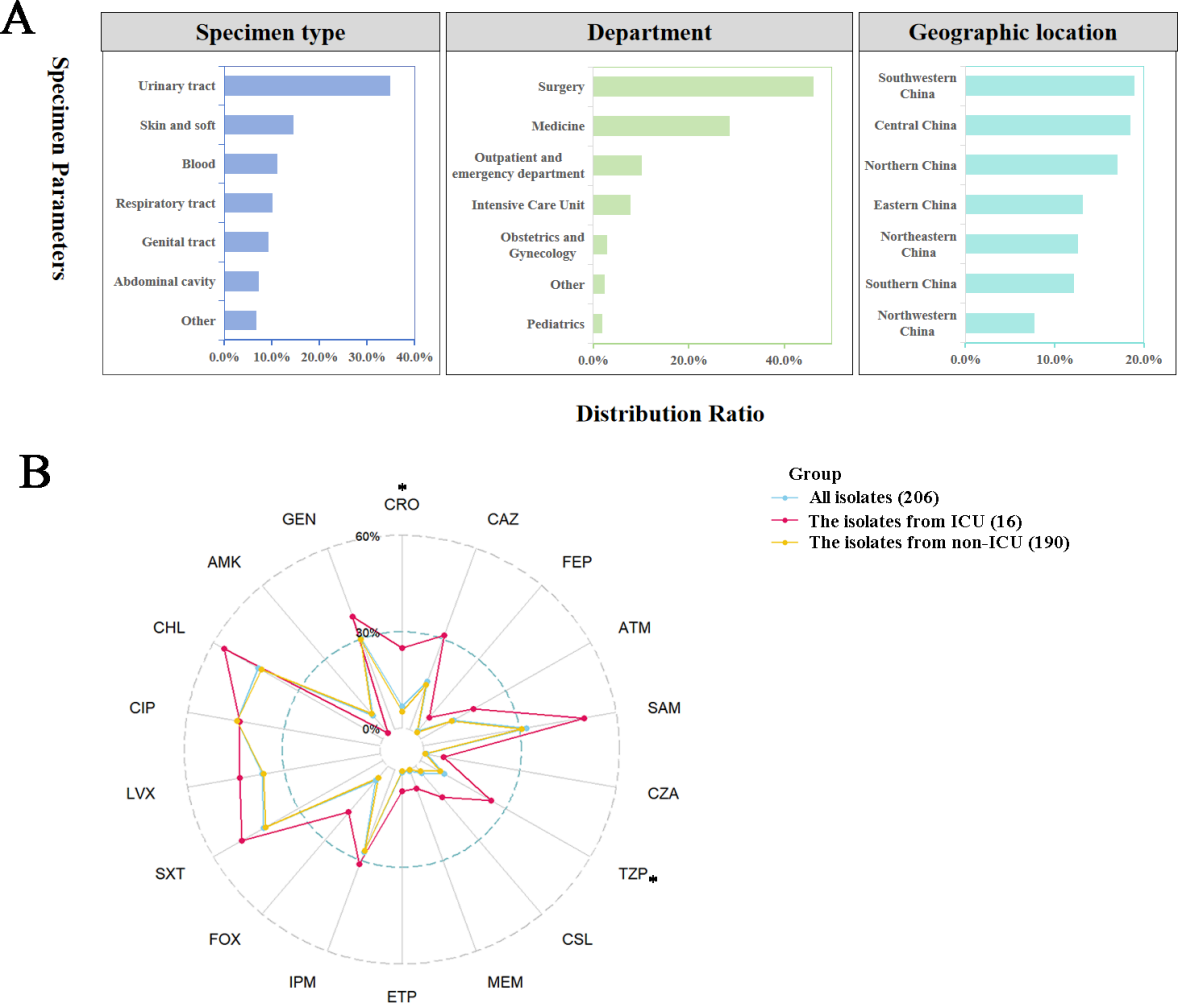
The origins information of 206 *Morganella morganii* isolates and their resistance rates to 18 antimicrobial agents. (A) Distribution of 206 *M*. *morganii* isolates based on specimen types, departments, and geographical locations. (B) The resistance rates of all *M. morganii*, the isolates from ICU and non-ICU to 18 antimicrobial agents. The numbers in the legend represent the number of isolates from the corresponding source. SAM, ampicillin-sulbactam; CRO, ceftriaxone; TZP, piperacillin-tazobactam; CAZ, ceftazidime; FEP, cefepime; ATM, aztreonam; SXT, trimethoprim-sulfamethoxazole; LVX, levofloxacin; FOX, cefoxitin; CIP, ciprofloxacin; CZA, ceftazidime-avibactam; CHL, chloramphenicol; AMK, amikacin; GEN, gentamicin; CSL, cefoperazone-sulbactam; MEM, meropenem; ETP, ertapenem; IPM, imipenem. * represented a significant difference (*P* < 0.05) in antimicrobial resistance rates between *M. morganii* isolated from the ICU and non-ICU.

Among the 206 *Morganella morganii* isolates, a considerable proportion (35.0%) were isolated from the urinary tract. Regarding distribution across hospital departments, the surgery and medicine wards accounted for 46.1% and 28.6% of the strains, respectively (Fig. 1A). The resistance rates of the 206 isolates to 18 tested antimicrobial agents are summarized in Fig. 1B. Overall, the strains demonstrated a low resistance rate, ranging from 0.5% to 16.0%, to amikacin and most tested β-lactams, with the exception of ampicillin-sulbactam (32.0%), imipenem (27.2%), trimethoprim-sulfamethoxazole (42.2%), levofloxacin (36.9%), ciprofloxacin (44.7%), chloramphenicol (44.2%), and gentamicin (30.6%) (Fig. 1B). No significant difference in resistance rates was observed among *M. morganii* isolates from different specimen types or geographic locations (data not shown). However, isolates from the Intensive Care Unit (ICU) exhibited significantly higher resistance rates to ceftriaxone (25.0% vs 5.3%, *P* < 0.05) and piperacillin-tazobactam (25.0% vs 6.9%, *P* < 0.05) compared to the isolates from non-ICU (Fig. 1B).

### Antibiotic resistome, virulence, and MGEs characteristics of *M. morganii*

The analysis of the antibiotic resistome of 431 *M*. *morganii* isolates (206 from this study and 225 from NCBI genome data sets) revealed a large range of resistance determinants, with 110 kinds of ARGs conferring resistance to 15 different categories of antimicrobial agents. The ARG count per isolate varied from 0 to 55, averaging 6.3 genes per isolate (Table S2). Notably, ARGs involved in resistance to multiple “last resort antimicrobial agents” including carbapenems (*bla*_KPC_, *bla*_NDM_, *bla*_IMP_, *bla*_GES_, and *bla*_OXA)_, polymixins (*mcr*), and tigecycline [*tet*(X4), and *tmexCD-toprJ*] were identified in *M. morganii* (Table S2). In parallel, a detailed screening of 133 virulence-related genes (VRG) profiles was conducted in the 431 *M*. *morganii* isolates. The vast majority of VRGs, such as genes related to motility-chemotaxis systems, urease hydrolysis and putrescine production, and fimbrial adhesins, were found to be nearly ubiquitous among the isolates (Tables S1 and S3). However, the *hlyCABD* operon, encoding the RTX toxin linked to bacterial hemolysis, was identified in only 29.0% (125/431) of the isolates (Table S3) (10). The analysis identified diverse MGEs in *M. morganii*. In addition to numerous insertion sequences and transposons, *M. morganii* also possesses a high load of genomic islands (averaging 4.9 per strain) and prophages (averaging 5.8 per strain) (Table S2). The *M. morganii* harboring genes, including ARGs, VRGs, and MGEs, were compared according to their sources (Tables 2 and 3; Table S4). The results suggest that there were no significant differences in the quantities of ARGs, VRGs, and MGEs carried by *M. morganii* collected from various geographic locations in this study. Compared to *M. morganii* from the NCBI genome data sets, those collected for this study exhibited a higher prevalence of VRGs but a lower prevalence of MGEs (*P* < 0.05). Considering that these differences might stem from the diverse host spectrum in the NCBI genome data sets, further analysis was conducted on the gene carriage among *M. morganii* from different host sources. The results revealed that *M. morganii* from Homo sapiens carried the most VRGs (*P* < 0.05).

### Emergence of a novel *bla*_KPC-2_-bearing plasmid in *M. morganii*

**Fig 2:**
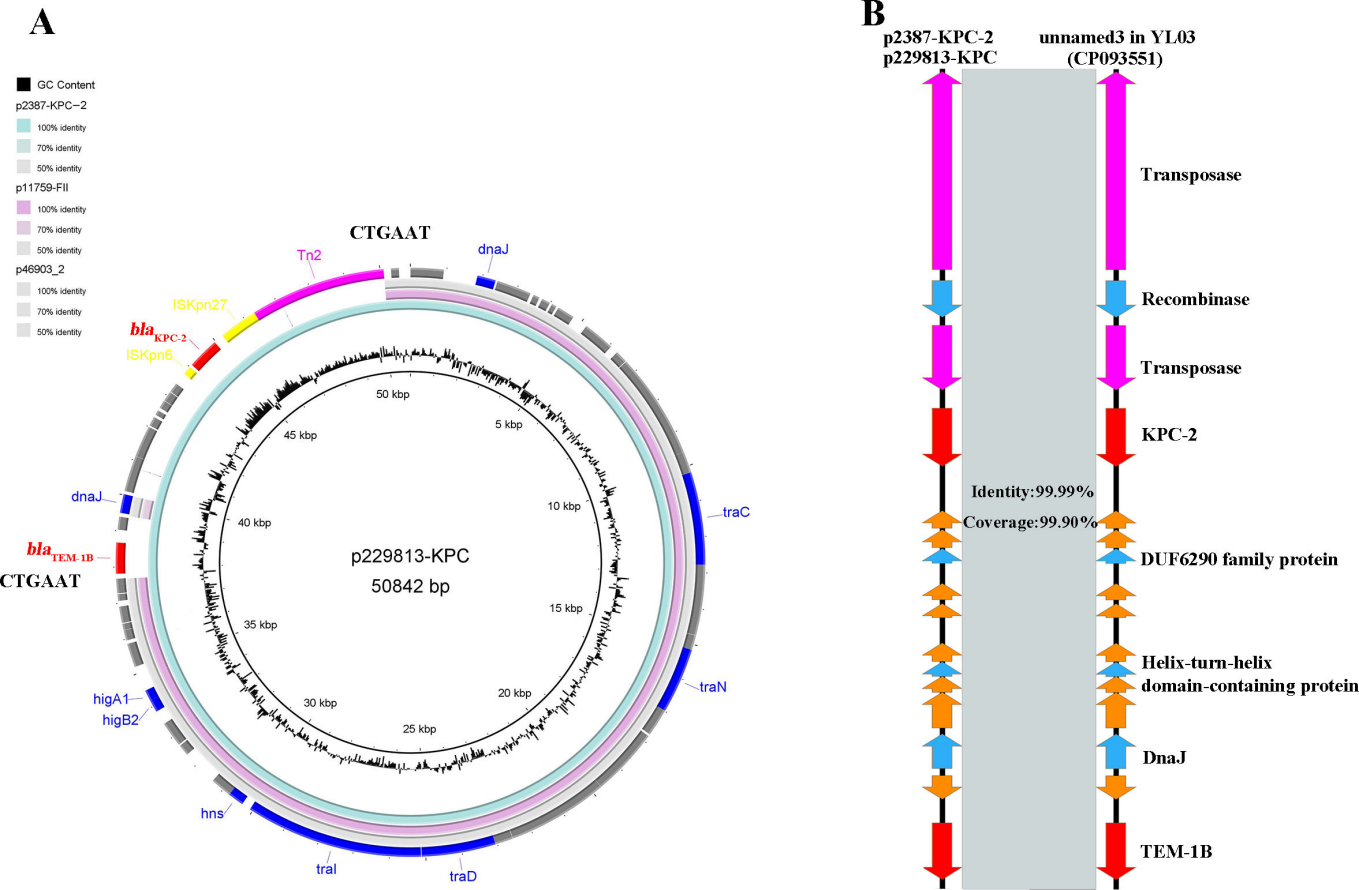
The possible sources of formation for the novel *bla*_KPC-2_-bearing plasmid in *M. morganii*. (A) The comparison between the novel *bla*_KPC-2_-bearing plasmids and their most similar plasmids. The internal ring was the reference sequence of the novel *bla*_KPC-2_-bearing plasmid p229813-KPC (MN310368.1). The outermost two rings were plasmids from *M. morganii* in the NCBI genome data sets, p11759-FII (MZ848139.1) and p46903_2 (CP070522.1), respectively. (B) Alignment of the genetic environment of transposition units carrying *bla*_KPC-2_. On the left was the transposon unit from the novel plasmids p2387-KPC-2 and p229813-KPC, and on the right was the corresponding matching transposon unit from the plasmid unnamed3 in *E. coli* YL03 (CP093551).

To further understand the dissemination of carbapenemase genes in *M. morganii*, a detailed analysis was conducted on 37 fragments containing carbapenemase genes (14 *bla*_KPC-2_, 13 *bla*_NDM-1_, and 10 other types). In the carbapenemase gene at known locations, aside from *bla*_IMP-27_ and two *bla*_NDM-1_ located in the chromosome, all remaining carbapenemase genes were found on plasmids (Table 1). This strongly suggests that horizontal transmission of plasmids was the primary driving factor behind the prevalence of carbapenemase genes in *M. morganii*. While almost all of these plasmids were commonly encountered in other Enterobacteriales (11), a novel *bla*_KPC-2_-harboring plasmid belongs to unknown Inc-type was identified in three isolates: 2387, GCF_018456265.1, and GCF_018456285.1, named separately as p2387-KPC-2 (CP139442.1), p229813-KPC (MN310368.1) and p516602-KPC (MN310367.1, linear and therefore not included in subsequent studies) (Table 1). When searched in the NCBI genome data sets, these plasmids were found to share a high degree of sequence homology with two *M. morganii*-hosted plasmids, p11759-FII (MZ848139.1) and p46903_2 (CP070522.1), neither of which contained carbapenemase genes. Compared to p11759-FII and p46903_2, the plasmids p2387-KPC-2 and p229813-KPC possessed an additional segment of 12,440 bp. This segment corresponded to the transposition unit featuring one transposase and two direct repeat sequences of CTGAAT (Fig. 2A). Upon further comparison, this transposition unit exhibited high similarity to a *bla*_KPC-2_-bearing fragment of *Escherichia coli* YL03 (CP093551) in the NCBI genome data sets (Fig. 2B). This finding suggested that the transposition unit carrying *bla*_KPC-2_ and *bla*_TEM-1B_ integrated into the *M. morganii*-hosted plasmid, resulting in the formation of a novel plasmid. Supporting this finding, the strain harboring p46903_2 was also found to possess another *bla*_KPC-2_-bearing plasmid, named p46903-KPC (Table 1). Furthermore, conjugation experiments confirmed the transferability of this novel *bla*_KPC-2_-bearing plasmid, with the conjugation frequency of 7.9*10^−5^ when transferred to the EC600 strain, suggesting its potential for horizontal transmission among Enterobacteriales.

**TABLE 1:** Characterization of 37 carbapenemase genes-bearing *Morganella morganii*

Carbapenemase genes	Isolate	Carbapenemase genes containing fragment	
		Location	Size
*bla*_KPC-2_			
	GCF_003071325.1	IncN plasmid	Circular, 71,304 bp
	GCF_016905845.1	IncR plasmid	Linear, 19,117 bp
	GCF_016939515.1	IncR plasmid	Circular, 54,351 bp
	GCF_016939575.1	IncP6 plasmid	Circular, 62,326 bp
	GCF_016939635.1	IncR plasmid	Circular, 54,347 bp
	GCF_017114545.1	IncR plasmid	Circular, 54,350 bp
	GCF_018456265.1	Unknown Inc-type plasmid	Circular, 50,842 bp
	GCF_018456285.1	Unknown Inc-type plasmid	Linear, 52,180 bp
	GCF_018475045.1	IncX6 plasmid	Linear, 46,166 bp
	GCF_018475595.1	IncX6 plasmid	Linear, 46,304 bp
	GCF_019656395.1	ColRNAI plasmid	Linear, 23,753 bp
	GCF_019656415.1	ColRNAI plasmid	Linear, 23,753 bp
	GCF_019656435.1	ColRNAI plasmid	Linear, 23,753 bp
	2387	Unknown Inc-type plasmid	Circular, 50,842 bp
*bla*_NDM-1_			
	24058	IncN plasmid	Linear, 9,269 bp
	70101	Unknown Inc-type plasmid	Linear, 8,620 bp
	GCF_000752335.1	Unknown Inc-type plasmid	Linear, 15,148 bp
	GCF_002588265.1	IncFIB-IncFII plasmid	Linear, 94,625 bp
	GCF_002968775.1	Tn3 is located in the chromosome	Circular, 4,139,887 bp
	GCF_003114875.2	IncFII-IncN plasmid	Linear, 12,877 bp
	GCF_003114875.2	IncN plasmid	Circular, 73,129 bp
	GCF_003852695.1	Unknown Inc-type plasmid	Linear, 11,430 bp
	GCF_016618235.1	*M. morganii* genomic island is located in the chromosome	Circular, 4,111,737 bp
	GCF_018475235.1	IncX3 plasmid	Linear, 44,363 bp
	GCF_018475355.1	IncX3 plasmid	Linear, 8,050 bp
	GCF_019052875.1	IncX3 plasmid	Circular, 44,962 bp
	GCF_021460795.1	Unknown	Linear, 4,301 bp
*bla*_NDM-5_			
	GCF_003955965.1	IncX3 plasmid	Circular, 4,6161 bp
*bla*_IMP-1_			
	GCF_014283905.1	Unknown	Linear, 880 bp
*bla*_IMP-27_			
	GCF_010365245.1	Tn7 located in the chromosome	Circular, 3,942,451 bp
	GCF_026627725.1	Tn7 located in the chromosome	Linear, 263,446 bp
	GCF_028867915.1	Tn7 located in the chromosome	Linear, 263,592 bp
	GCF_030164825.1	Tn7 located in the chromosome	Linear, 47,246 bp
*bla*_GES-5_			
	GCF_002029935.1	Unknown	Linear, 1,486 bp
*bla*_OXA-181_			
	GCF_000770295.1	ColKP3 plasmid	Linear, 13,940 bp
	GCF_014333515.2	ColKP3 plasmid	Linear, 8,567 bp
*bla*_OXA-48_			
	GCF_030177045.1	IncL plasmid	Linear, 65,489 bp

### Emergence of a genospecies with abundant flagellar-related genes in *M. morganii* subsp. *sibonii* based on genomic classification

**Fig 3:**
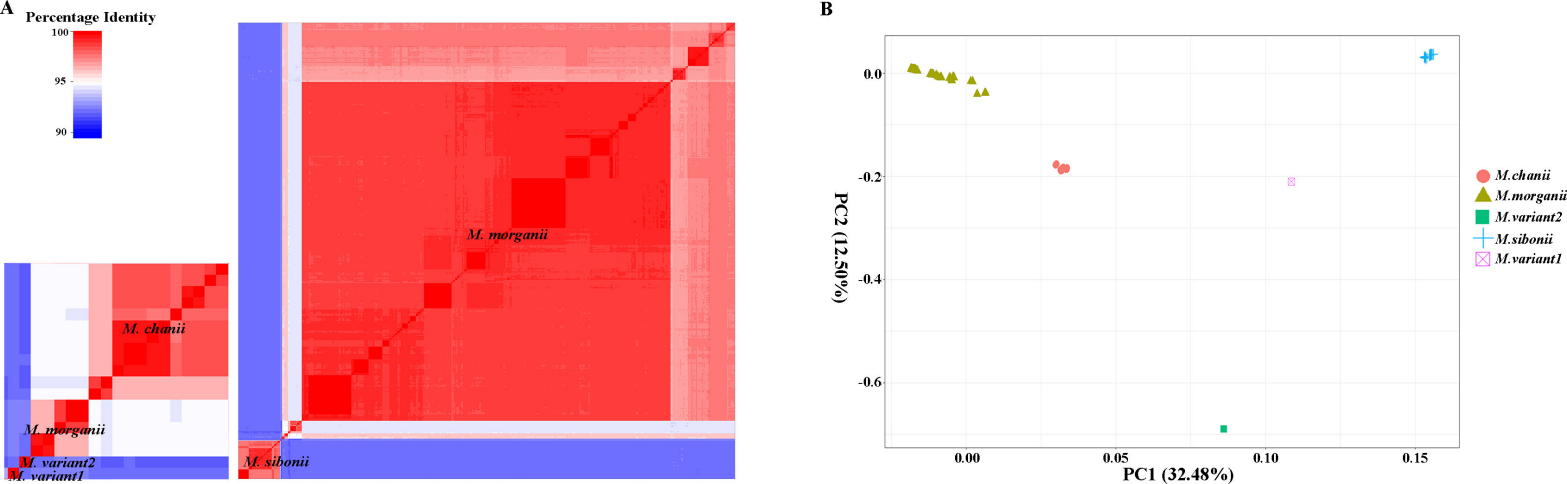
The genomic classification of 431 *M*. *morganii* isolates. (A) Pairwise average nucleotide (ANI) identity comparison was calculated for all *M. morganii* isolates shown on a heatmap with blue indicating low and red indicating high nucleotide identity. The left diagram was an enlargement of a portion of the right diagram. (B) Principal component analysis (PCA) plot of the different genospecies of *M. morganii*.

To conduct the genomic classification of *M. morganii*, we calculated pairwise average nucleotide identity (ANI) values for 431 isolates. All *M. morganii* isolates were classified into five distinct genospecies, adhering to the 95% ANI threshold for genospecies delineation (Fig. 3A) (12). Based on the previous research (7), these genospecies were designated as *M. morganii* (381 isolates), *M. sibonii* (36 isolates), *M. chanii* (12 isolates), *M. variant1* (one isolate, corresponding to *M. laugraudii* in the previous research), and *M. variant2* (one isolate, not identified in the previous research). In addition, a principal component analysis (PCA) plot similarly indicated that the 431 *M*. *morganii* could be categorized into five distinct clusters (Fig. 3B). To comprehend the distinctions in the trehalose operon (*treRBP*) among different genospecies excluding *M. morganii* and one *M. chanii* genospecies without *treRBP*, a total of 49 *treRBP* sequences were extracted for multiple sequence alignment and phylogenetic tree construction. The findings unveiled that the *treRBP* within the identical genospecies were categorized into corresponding clusters, consistent with prior ANI and PCA analyses (Fig. S1). Despite the sequence variations observed in the *treRBP* genes across different genospecies, all retained the functional capability to ferment trehalose. Thus, *M. sibonii*, *M. chanii*, *M. variant1,* and *M. variant2* genospecies belonged to *M. morganii* subsp. *sibonii*, and *M. morganii* genospecies fall under *M. morganii* subsp. *Morganii*, according to previous classification (13).

**Fig 4:**
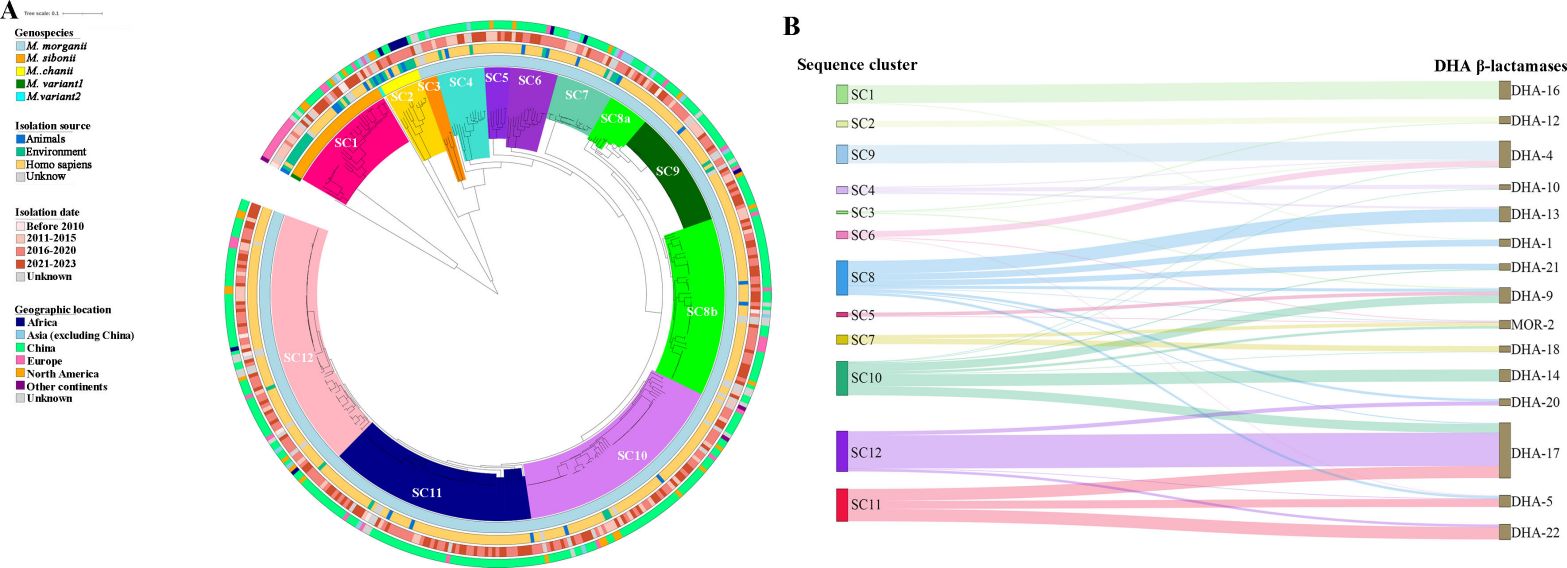
Phylogenetic analysis of 431 *M*. *morganii* isolates. (A) The phylogenetic tree of *M. morganii* isolates with their sequence clusters and corresponding background information. (B) Types of DHA β-lactamases produced by different sequence clusters of *M. morganii*.

Building upon the background information of 431 isolates, we conducted a detailed analysis of the epidemiological and molecular characteristics of different *M. morganii* genospecies. While the *M. morganii* genospecies predominantly originated from Homo sapiens (87.9%), a significantly larger proportion of *M. sibonii* and *M. chanii* strains were derived from environmental sources, accounting for 38.9% and 50%, respectively. There was no significant variation in the geographical and temporal distributions of the different genospecies (Fig. 4A). Leaving aside *M. variant1* and *M. variant2* genospecies, which had only one isolate each, *M. morganii* genospecies exhibited the highest load of ARGs (averaging 6.7 per strain) and VRGs (averaging 130.0 per strain), while *M. chanii* genospecies displayed the fewest of these genes (averaging 1.4 ARGs and 120.7 VRGs per strain) (*P* < 0.05). Interestingly, *M. chanii* genospecies harbored an equivalent amount of MGEs (averaging 18.6 per strain) to that of *M. morganii* and *sibonii* (averaging 17.0 and 18.6 per strain, respectively) (Fig. S2A). A comparative genomic analysis between *M. chanii* and *M. sibonii* genospecies revealed a notable abundance of cell motility genes, primarily flagellar-related and predominantly located within MGEs, in the *M. chanii* genospecies (Table S5). Contrastingly, *M. variant1* and *M. variant2* genospecies harbored minimal ARGs (0 and 1, respectively) and VRGs (112 and 111, respectively), significantly lower than the average counts (6.3 of ARGs and 129.0 of VRGs) (Table S2). In stark contrast, they possessed 25 and 27 MGEs, respectively, with particular enrichment in genomic islands (14 in *M. variant2*) and prophages (eight for both *M. variant1* and *M. variant2*) (Table S2). Therefore, *M. variant1* and *M. variant2* genospecies demonstrated a greater number of genes related to replication, recombination, repair, and transcription compared to *M. sibonii* and *M. chanii* (Tables S6 and S7).

### Emergence of a sequence cluster enriched with ARGs, VRGs, and MGEs in *M. morganii subsp. morganii* based on phylogenomic analysis

For further phylogenomic analysis of the *M. morganii*, a maximum likelihood phylogeny was constructed based on 70311 filtered cgSNPs identified from 431 *M*. *morganii* sequences. The rhierBAPS population structure analysis clustered the 431 genomes into 12 distinct sequence clusters (SCs), denoted as SC1 through SC12. As expected, *M. sibonii* and *M. chanii* genospecies were divided into two SCs, SC1 and SC2, respectively (Fig. 4A). The *M. variant1* and *M. variant2*, each comprising only one isolate, have not been categorized into any of the identified SCs. The presence of *M. morganii* from varied sources, geographic locations, and time points within the same SC suggested a widespread propagation capability of the strain across different hosts and regions. The distribution of sequence clusters among *M. morganii* from various geographic locations in this study shows no significant differences (Table S4). However, the distribution of sequence clusters SC1, SC2, and SC8 varied significantly among *M. morganii* from different hosts (Table 2, *P* < 0.05). In addition, significant differences were observed in the distribution of SC1, SC7, and SC11 between isolates from the NCBI genome data sets and this study (Table 3, *P* < 0.05). A noteworthy revelation lay in the strong correlation observed between the sequence cluster of *M. morganii* and the specific β-lactamase DHA (or MOR) types it inherently harbored (Fig. 4B). Within multiple SCs, the majority of strains predominantly produced a specific DHA type, for instance, SC1 strains predominantly featured DHA-16, while SC9 strains were characterized by DHA-4 production.

**TABLE 2:** The molecular epidemiology among the *Morganella morganii* collected from various host

Category		Animals	Environment	*Homo sapiens*
Number of genes carried				
	Number of antimicrobial resistance genes	6.5	3.9	6.4
	Number of mobile genetic elements	19.9	18.4	16.8
	Number of virulence-related genes^*a*^	123.7	124.2	130.2
The proportion of sequence cluster				
	SC1^*a*^	14.3%	42.4%	4.5%
	SC2^*a*^	14.3%	18.2%	0.8%
	SC3	0.0%	0.0%	0.8%
	SC4	4.8%	6.1%	3.4%
	SC5	0.0%	0.0%	2.3%
	SC6	9.5%	3.0%	3.4%
	SC7	9.5%	9.1%	3.4%
	SC8^*a*^	9.5%	0.0%	17.2%
	SC9	4.8%	6.1%	9.3%
	SC10	9.5%	6.1%	17.2%
	SC11	14.3%	3.0%	16.7%
	SC12	0.0%	6.1%	20.9%

^*a*^ Represented a *P*-value of <0.05.

**TABLE 3:** The molecular epidemiology between the two groups of *Morganella morganii* from NCBI genome data sets and this study

Category		NCBI	This study
Number of genes carried			
	Number of antimicrobial resistance genes	6.8	5.8
	Number of mobile genetic elements^*a*^	18.3	16.0
	Number of virulence-related genes^*a*^	128.2	130.4
The proportion of sequence cluster			
	SC1^*a*^	11.6%	4.9%
	SC2	4.0%	1.5%
	SC3	0.9%	1.5%
	SC4	4.9%	1.9%
	SC5	1.3%	2.4%
	SC6	3.6%	3.4%
	SC7^*a*^	6.2%	1.9%
	SC8	14.2%	18.0%
	SC9	10.2%	6.8%
	SC10	15.6%	16.0%
	SC11^*a*^	10.7%	19.9%
	SC12	16.0%	21.8%

^*a*^ Represented a *P*-value of <0.05.

**Fig 5:**
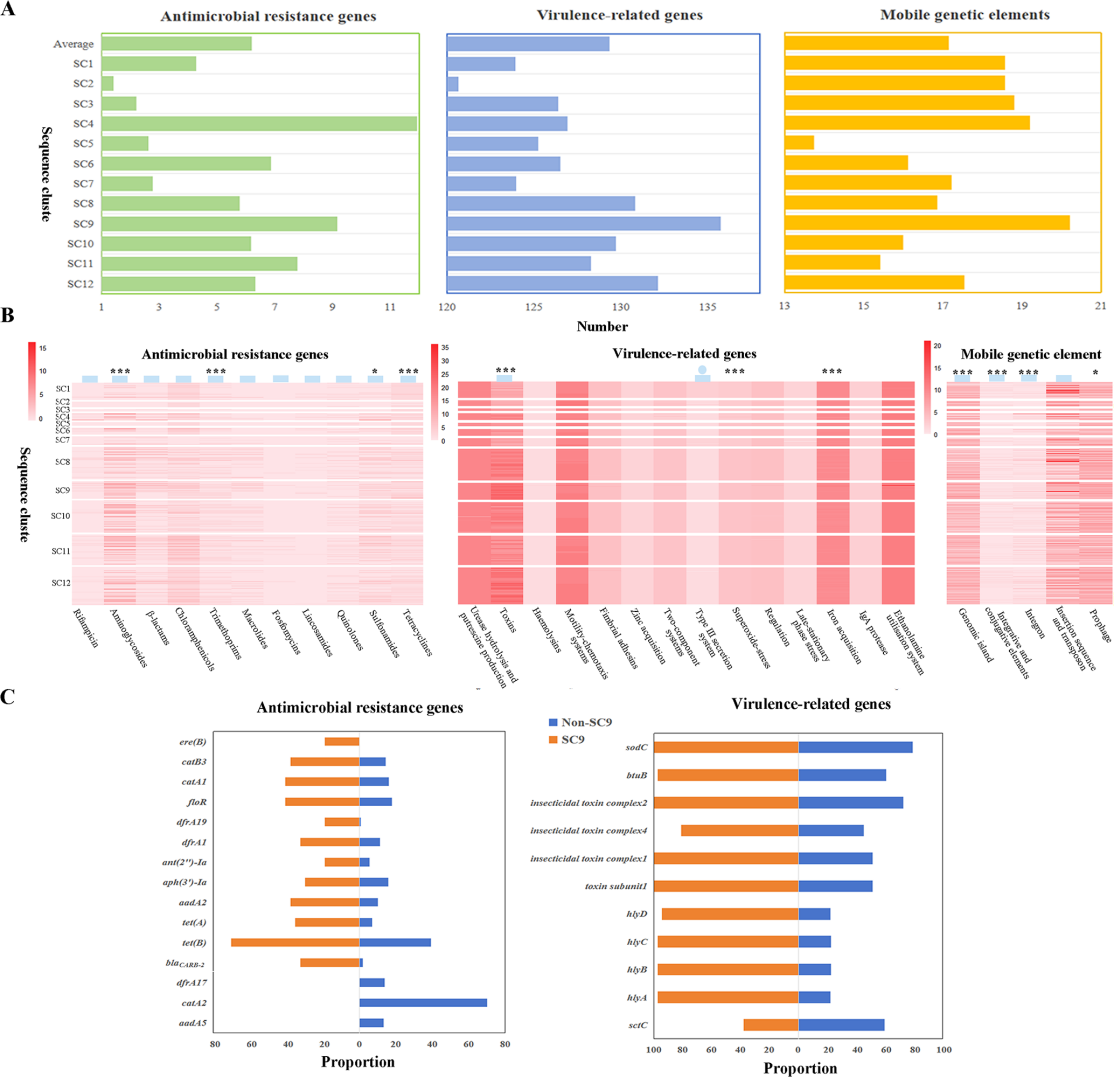
The analysis of the antimicrobial resistance genes (ARGs), virulence-related genes (VRGs), and mobile genetic elements (MGEs) carried by different sequence clusters of *M. morganii*. (A) Variation in the quantity of ARGs, VRGs, and MGEs among different sequence clusters of *M. morganii*. (B) Profiles of ARGs, VRGs, and MGEs carried by different sequence clusters of *M. morganii*. * indicates that SC9 strains carry a significantly higher number of the corresponding category compared to non-SC9 strains (* represented a *P*-value of <0.05, and *** represented *P* < 0.01), and the circle represented non-SC9 strains carried more of the corresponding category. The box represents a high degree of dispersion in the number of resistance genes carried by different SCs (coefficient of variation greater than 15%). (C). Distribution of ARGs and VRGs with significant differences between SC9 and non-SC9 strains.

A comprehensive analysis was undertaken to assess the abundance of ARGs, VRGs, and MGEs within each SC of *M. morganii*. Overall, different categories of ARGs and MGEs, excluding prophage, exhibit significant variations across distinct clusters with a coefficient of variation greater than 15%. By contrast, VRGs displayed a relatively homogeneous distribution across the clusters, apart from genes related to toxins and the type III secretion system (Fig. 5B). Notably, one specific sequence cluster, SC9, classified under *M. morganii* genospecies, stood out with the highest average counts of VRGs and MGEs, and ranked second in ARG abundance, only surpassed by SC4 (Fig. 5A). The isolate GCF_016618235.1 in SC4 harbored 55 ARGs due to gene copies, elevating the average ARG count in SC4 (14). The ARGs, VRGs, and MGEs in SC9 strains were significantly more abundant than those in non-SC9 strains (*P* < 0.01, Fig. S2B). Focusing on ARGs, SC9 strains carried more genes conferring resistance to aminoglycosides, trimethoprims, sulfonamides, and tetracyclines than non-SC9 strains (*P* < 0.05, Fig. 5B). Specifically, the prevalence of 12 genes such as *bla_CARB-2_*, *tet(B) and tet(A*) was higher in SC9 strains, while *dfrA17*, *catA2,* and *aadA5* exhibited higher prevalence in non-SC9 strains (*P* < 0.05, Fig. 5C). In the realm of VRGs, SC9 strains displayed a marked disparity in toxin-related genes compared to non-SC9 strains, especially *hlyCABD* encoding RTX toxin (15). These gene clusters were present in 97.3% of SC9 strains and significantly less prevalent in 22.6% of non-SC9 strains (Fig. 5C). In addition, the abundance of *sodC* (related to superoxide-stress) and *btuB* (involved in iron acquisition) was significantly higher in SC9 strains, while *sctC* (associated with type III secretion system) was more prevalent in non-SC9 strains (Fig. 5C). Except for insertion sequences and transposons, the number of other MGEs in SC9 strains was significantly higher than in non-SC9 strains.

## DISCUSSION

MGEs, such as genomic islands (GIs), integrative conjugative elements (ICEs), transposes (Tns), insertion sequences (ISs), and prophages, were instrumental in markedly shaping bacterial genome structures (16). These elements facilitated the exchange of genetic material, thus playing a pivotal role in the evolution of bacterial strains (17). Prior research has identified the high prevalence of various MGEs in *M. morganii*, underlining their significant role in enhancing genomic plasticity within this species (7, 14, 18, 19). In line with previous reports (2, 7), our study detected five distinct genospecies within the *M. morganii* at the genome scale, surpassing two clusters that correspond to the two conventional subspecies. In addition, sequence variations in *treRBP* genes were observed among these genospecies of *M. morganii* subsp. *sibonii*, suggesting evolutionary divergence within the subspecies with a substantial proportion of isolates originating from the environment. Despite abundant MGEs, *M. chanii* genospecies demonstrated a notably lower prevalence of ARGs and VRGs compared to *M. sibonii* genospecies. Comparative genomic analysis reveals that *M. chanii* genospecies carries a greater number of flagellar-related genes, primarily located within MGEs (Table S5). Considering that flagellar-related genes contributed to movement toward favorable environments and that environmental complexity played a crucial role in strain evolution (20, 21), the presence of a diverse array of MGEs in *M. chanii* genospecies might facilitate the adaptive evolution in response to environmental challenges rather than an augmented repertoire of resistance and virulence determinants. Unlike *M. chanii*, *M. variant1* and *M. variant2* genospecies were found to lack a substantial number of flagellar-related genes. Instead, these genospecies were characterized by a significant presence of genes related to replication, recombination, repair, and transcription, which was attributed to their abundant GIs and prophages (Tables S6 and S7). Further research is warranted to determine whether these genomic differences manifest as distinct phenotypic characteristics.

Unlike *M. morganii* subsp. *sibonii*, which was primarily isolated from environmental sources, *M. morganii* subsp. *morganii* was predominantly isolated from Homo sapiens, and its evolutionary trajectory was significantly influenced by the selective pressure of antimicrobial agents. Phylogenomic analysis, integrated with molecular epidemiology, revealed that SC9 strains belonging to *M. morganii subsp. morganii*, possessed a higher number of ARGs compared to non-SC9 strains, mainly including genes conferring resistance to aminoglycosides, trimethoprims, sulfonamides, and tetracyclines, consistent with previous results (19). This discrepancy was particularly evident for aminoglycoside resistance genes. On average, SC9 strains contained 2.4 aminoglycoside resistance genes per strain, in contrast to 1.4 in non-SC9 strains. Specifically, a significantly higher prevalence of the *aadA2*, *aph(3')-Ia*, and *ant(2'')-Ia* genes was noted in SC9 strains. In addition, both this study and previous reports consistently demonstrate aminoglycoside resistance genes were frequently associated with MGEs (22), and SC9 strains harbor more MGEs compared to non-SC9 strains. Given the clinical preference for cephalosporins and aminoglycosides in treating *M. morganii* infections (6), we propose that the selection pressure from aminoglycosides and other antimicrobial agents along with the accumulation of MGEs could be one of the vital factors for the emergence and epidemic success of this sequence cluster. Furthermore, it is worth noting the near-universal presence of the *hlyCABD* gene cluster encoding the RTX toxin in SC9 strains, potentially enhancing their virulence compared to non-SC9 strains. Therefore, particular attention should be paid to the epidemiological development of the SC9 strains. In addition, for the first time, we discovered an association with the sequence cluster of *M. morganii* and its intrinsic resistance gene *bla*_DHA_ type. The observation of similar phenomena has also been made within the *Klebsiella oxytoca* complex where sequence variations in the chromosomally-encoded β-lactamase gene, *bla*_OXY_, categorized the bacteria into different phylogroups (23). Considering *M. morganii* lacks MLST typing, the *bla*_DHA_ gene may provide a foundational element for developing a gene-based typing system.

It is known that interplasmid horizontal transfer mediated by MGEs, such as transposons, plays a critical role in the emergence of novel plasmids. One illustrative example was the generation of conjugative plasmids simultaneously encoding for carbapenem resistance and hypervirulence. These plasmids contained a wide range of transposable elements that enabled them to undergo frequent genetic transposition (24). Kayoko Sugita elaborated on the interplasmid transposition of *bla*_KPC-2_-containing Tn4401a from an IncN+R plasmid to a ColRNAI plasmid in *Klebsiella pneumoniae* (25). In this study, while the detection rate of the carbapenemase genes in *M. morganii* was relatively low, we found that most of these genes were harbored within plasmids. Notably, we also identified a novel plasmid carrying *bla*_KPC-2_, which is inserted into *M. morganii*-specific plasmids by a transposon derived from *E. coli*. The identified plasmid, possessing conjugative transfer capability along with the carried ARGs, may contribute to the further dissemination of KPC-2-producing *M. morganii*.

### Conclusions

In conclusion, our data indicate the significant contribution of MGEs in the evolutionary trajectory of two *M. morganii* subspecies. In *M. morganii* subsp. *sibonii*, typically isolated from environmental sources, the evolution of *M. chanii* genospecies has occurred, enhancing adaptation to the environment. In *M. morganii* subsp. *morganii*, mainly isolated from Homo sapiens, the SC9 strains, characterized by an increased abundance of ARGs and VRGs, might evolve into highly successful clones and pose a burgeoning challenge in healthcare settings. In addition, the novel *bla*_KPC-2_-bearing plasmids, formed under the action of MGEs, may promote the spread of carbapenemase in *M. morganii*. These observations underscore the critical importance of vigilant surveillance of *M. morganii* prevalence to prevent its emergence as a formidable obstacle in clinical therapeutics.

## MATERIALS AND METHODS

### Bacterial isolates and identification

This study utilized a comprehensive surveillance program, selecting the clinical *Morganella morganii* isolates based on stringent criteria: the host had a clinical diagnosis indicating a potential infection, and only a single isolate per patient was included. Under this standard, a total of 206 *M*. *morganii* isolates were gathered from 45 hospitals across 26 provinces and municipalities that cover seven regions of China: northwestern China (*n* = 16), southern China (*n* = 25), northeastern China (*n* = 26), eastern China (*n* = 27), northern China (*n* = 35), central China (*n* = 38), and southwestern China (*n* = 39). The isolates were obtained from various specimens and hospital departments, as illustrated in Fig. 1A. Identification of all *M. morganii* isolates was performed using matrix-assisted laser desorption ionization-time of flight (MALDI-TOF) mass spectrometry.

### Antimicrobial susceptibility testing

The bacterial antimicrobial susceptibility was tested using the broth microdilution method followed by Clinical and Laboratory Standards Institute (CLSI) standards (26). The susceptible breakpoints of cefoperazone defined by the CLSI were applied for cefoperazone-sulbactam. The susceptibilities of the 17 remaining antimicrobial agents were interpreted according to current CLSI guidelines (27).

### Whole-genome data sequencing and genome analysis

The genomic DNA of all 206 isolates was subjected to draft-genome sequencing using a paired-end library on illumina novaseq 6000 system, and isolate 2387 was complete-genome sequenced using PacBio RSII sequencer. After being removed low-quality sequences and adapters, the reads were *de novo* assembled by the SPAdes Genome Assembler (v3.11.1) and hybrid assembled using Unicycler (v0.4.6) (28, 29). To enrich the analyzed data, we also retrieved a total of 225 reference genomes of *M. morganii* from NCBI genome data sets as of July 2023. Antibiotic resistome was characterized with abricate (v1.0.1) (30) screened against the ResFinder database (31). Due to the absence of virulence-related genes (VRGs) of *M. morganii* in the Virulence Factors of Pathogenic Bacteria Database (VFDB) (32), we established a list of virulence-related genes (the included virulence factors are listed in Table S1) for *M. morganii* based on the *M. morganii* KT strain (8) and the study by Palmieri et al. (13) and used BLASTx (v2.5.0) analysis for the detection of VRGs through this list (33). Plasmid types were identified using PlasmidFinder (v2.1) available at the Center for Genomic Epidemiology (CGE) (34). Comparisons of sequences from the novel *bla*_KPC-2_-bearing plasmid discovered in this study were performed by BRIG (v0.95) (35), and Easyfig (v2.2.3) was used to visualize the linear alignment of the genetic structure of the transposition unit carrying *bla*_KPC-2_ (36). The set of mobile genetic elements (MGEs) including genomic islands (GIs), integrative conjugative elements (ICEs), integron, prophage, transposes (Tns), and insertion sequences (ISs) in all *M. morganii* were performed utilizing Mobilome Prediction at VRprofile2 database (37).

### Phylogenetic analysis

The pairwise average nucleotide identity (ANI) values of *M. morganii* genome sequences were calculated using pyANI (v0.2.12) (38). The principal component analysis (PCA) was performed in Plink (v1.9) and R (v4.1) based on *M. morganii* genome sequences (39). Phylogenetic analysis was performed by constructing an assembly-based core-genome single nucleotide polymorphisms (SNPs) alignment using parsnp (v1.7.4) from the Harvest suite, incorporating recombination detection (40). Based on the recombination-free core-genome SNPs, a maximum-likelihood phylogenetic tree was constructed using RAxML (v0.6.0) with GTR + Gamma model, and 100 bootstrap replicates (41). iTOL (v3.0) was employed for annotating the tree combining the background information of the strain (42). The sequence clusters within the tree were identified through a hierarchical Bayesian Analysis of Population Structure (hierBAPS) model with a maximum depth of 2 and a maximum population number of 20 (43). To investigate the phylogeny of the trehalose operon (*treR*, *treB*, and *treP*), sequences from 36 *M*. *morganii* isolates were extracted using the seqkit (v2.3.0) (44), and were utilized for phylogenetic tree construction in MEGA X (45).

### Comparative genomic analysis

The assemblies of *M. morganii* were annotated with prokka (46) (v1.14.6), and the pan-genome was calculated using roary (47) (v3.13.0). Scoary (48) (v1.6.16) was employed to compare the differential genes between various genospecies of *M. morganii subsp. sibonii* with both sensitivity and specificity of ≥90%. The identified differential genes were annotated and analyzed using eggNOG-mapper (49) (v2).

### Conjugation experiments

The conjugation experiment was performed by the filter-mating method, employing the rifampin-resistant *Escherichia coli* EC600 as the recipient and *bla*_KPC-2_-bearing isolate 2387 as the donor strain. In brief, isolate 2387 and the EC600 were separately cultured in Luria-Bertani (LB) broth at 37°C for 4 hours. Subsequently, the mixture was incubated on Mueller-Hinton (MH) agar plates with 1 µg/mL meropenem and 600 µg/mL rifampin to select the transconjugant. The conjugation frequency was calculated as the number of transconjugants divided by donors. All experiments were carried out three times.

### Trehalose fermentation

Strains selected for this study included those carrying *treR*, *treB*, *and treP*, along with a negative control strain devoid of these genes. Bacterial suspensions were adjusted to a 0.5 McFarland standard and subsequently analyzed using the VITEK-2 system.

### Statistical analysis

The Mann-Whitney U rank sum test was applied to pairwise group comparisons of ARGs, VRGs, and MGEs. Pearson’s chi-square test or Fisher’s exact test was applied to evaluate the differences in antimicrobial resistance rates, ARGs, VRGs, and MGEs prevalence among the *M. morganii. P* values < 0.05 were considered statistically significant. All the statistical analyses were performed by Statistical Package for the Social Sciences (SPSS, v24.0).

## Data Availability

All assembled sequence data have been deposited in GenBank under the BioProject accession number PRJNA1042612. Individual accession numbers are also available in Table S2.

## SUPPLEMENTAL MATERIAL

Supplemental figures msystems.00306-24-s0001.docx Figures S1 and S2.

Supplemental tables msystems.00306-24-s0002.xlsx Tables S1 to S7.

ASM does not own the copyrights to Supplemental Material that may be linked to, or accessed through, an article. The authors have granted ASM a non-exclusive, world-wide license to publish the Supplemental Material files. Please contact the corresponding author directly for reuse.
