# Supplementary material for: Tracing the possible evolutionary trends of Morganella morganii: insights from molecular epidemiology and phylogenetic analysis
Source: mSystems. 2024 Jun 17;9(7):e00306-24. doi: 10.1128/msystems.00306-24 (PMC11264931; doi:10.1128/msystems.00306-24)
Supplement: Supplemental figures — Figures S1 and S2. [file msystems.00306-24-s0001.docx]

**Supplementary materials**


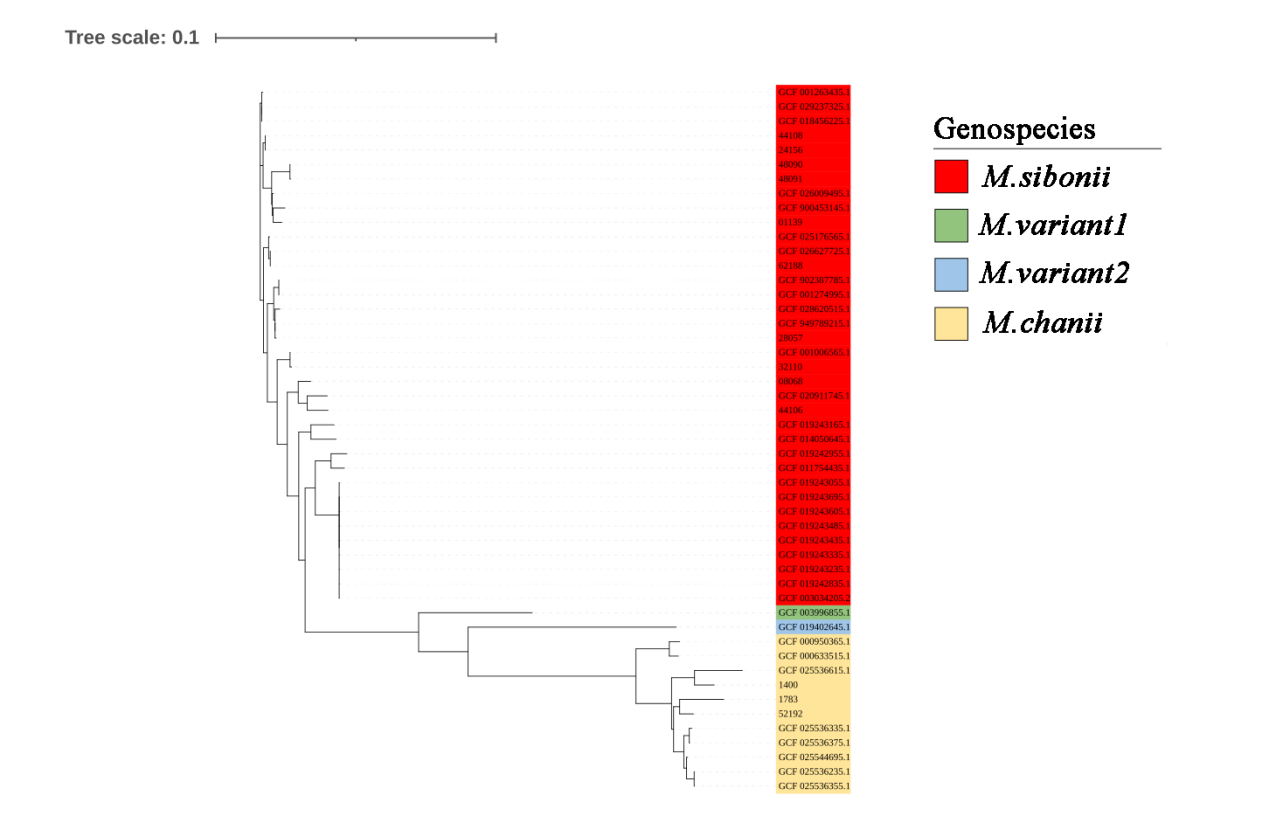


**Figure S1.** Phylogeny of trehalose operon (*treR*, *treB*, and *treP*) from 49 *M. morganii* isolates in generated by the maximum likelihood method


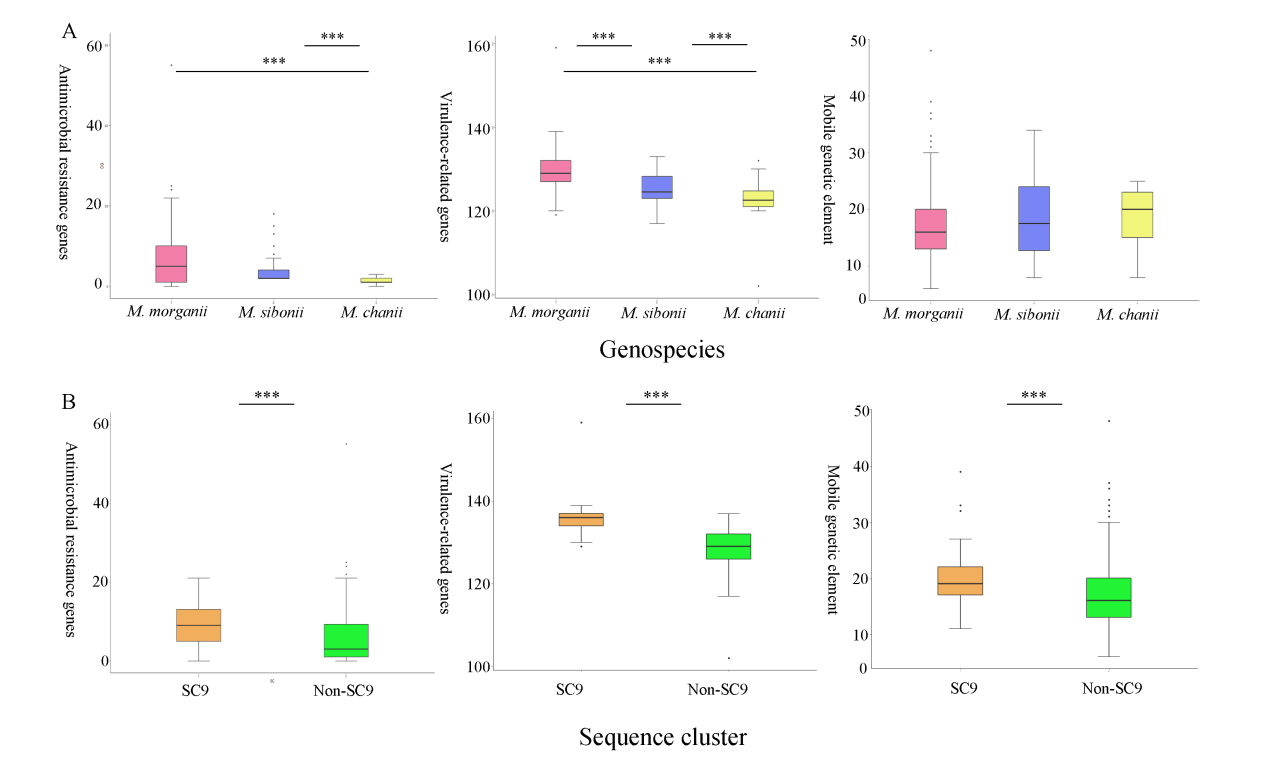


**Figure S2.** Box plot of ARGs, VRGs and MGEs distribution across different groups of *M. morganii.* *** represented a significant differences (*P* <0.01) in characteristics between two groups. **A.** Comparison of the number of ARGs, VRGs and MGEs carried by different genospecies of *M. morganii.* **B.** Comparison of the number of ARGs, VRGs and MGEs carried by SC9 strains and Non-SC9 strains.
